# Combined Ventricular Assist Device Placement With Adjustable Gastric Band (VAD-BAND): A Promising New Technique for Morbidly Obese Patients Awaiting Potential Cardiac Transplantation

**DOI:** 10.4021/jocmr814w

**Published:** 2012-03-23

**Authors:** Richdeep S. Gill, Shahzeer Karmali, Jeevan Nagandran, Howard O. Frazier, Vadim Sherman

**Affiliations:** a Department of Surgery, University of Alberta, Edmonton, Alberta, Canada; b Center for the Advancement of Minimally Invasive Surgery (CAMIS), Royal Alexandria Hospital, Edmonton, Alberta, Canada; c Michael E. DeBakey Deparment of Surgery, Division of Cardio-thoracic Surgery, Baylor College of Medicine, Houston, Texas, USA; d Michael E. DeBakey Department of Surgery, Division of General Surgery, Baylor College of Medicine, Houston, Texas, USA; e Richdeep S. Gill and Shahzeer Karmali were co-first authors.

## Abstract

**Background:**

Morbid obesity remains a potential relative contraindication for cardiac transplantation. Hence, a select population of morbidly obese patients with end-stage heart failure may require a ventricular assist device (VAD) as a bridge to transplantation to afford them time to lose sufficient weight and thus decrease there mortality rate after transplantation. Unfortunately, obtaining suitable weight loss via dietary or exercise regimens is limited by the cardiac limitations of the patients. We report on a new procedure of combining ventricular assist device placement with adjustable gastric band (VAD-BAND) placement to facilitate sufficient weight loss for cardiac transplantation.

**Methods:**

We report on our experience of 2 morbidly obese (BMI 46.6, BMI 43.7) patients with severe non-ischemic cardiomyopathy who underwent a VAD-BAND placement for treatment of morbid obesity and potential future cardiac transplantation.

**Results:**

Patient 1 was a 24-year-old male with a body mass index (BMI) of 46.6 admitted in cardiogenic shock with severe non-ischemic cardiomyopathy (New York Functional Class IV, Left Ventricular Ejection Fraction 15.3%) who underwent the VAD-BAND procedure. At 11 months outpatient follow-up the patient had clinically improved with a BMI of 34.2. Patient 2 was a 36-year-old male with a body mass index of 43.7 admitted in cardiogenic shock with severe non-ischemic cardiomyopathy (New York Functional Class IV, Left Ventricular Ejection Fraction 17.1%) who underwent placement of a VAD-BAND. At 4 months post-operation, the patient was stalwart clinically with a BMI of 34.8. Both patients are now under consideration for cardiac transplantation.

**Conclusion:**

In conclusion, concurrent placement of a VAD-BAND is a safe and viable option for morbidly obese patients with end-stage heart disease. Further research is needed to define indications and future clinical practice.

**Keywords:**

Gastric band; Ventricular assist device; Morbid obesity; Heart failure; Transplantation; Weight loss

## Introduction

According to the World Health Organization, there are over 500 million obese individuals worldwide classified as obese (BMI ≥ 30 kg/m^2^) [1], with a significant proportion of these individuals classified as morbidly obese (BMI ≥ 40 mg/kg^2^). Americans have been reported to have highest BMI of developed countries with approximately 70% of the adult population classified as either overweight or obese [2, 3]. In Canada, approximately 60% of the adult population is classified as either overweight or obese [4]. In addition, the obesity epidemic continues to increase and is associated with development of obesity-related comorbidities. Concurrently, in the US, heart failure affects over 5.5 million patients [5]. Recently, Kenchaiah et al reported an 11% increase in heart failure risk among men with elevated BMI compared to those with normal BMI [6]. In addition, morbid obesity is a relative contraindication to heart transplant. Grady et al reported an increased mortality rate following heart transplantation in patients > 140% of ideal body weight [7]. Hence, many morbidly obese transplant patients require ventricular assist devices to bridge them until they have lost sufficient weight to be considered for cardiac transplantation. Unfortunately, obtaining suitable weight loss via dietary or exercise regimens is limited by the functional limitations of patients with end-stage heart failure and has been shown to be of limited success. We report on a new procedure of combining ventricular assist device placement with adjustable gastric band (VAD-BAND) placement to facilitate sufficient weight loss for possible future consideration of cardiac transplantation.

## Methods

We report on our experience of two morbidly obese (BMI 46.6, BMI 43.7) patients with severe non-ischemic cardiomyopathy who underwent a combined placement of Ventricular Assist Device and Adjustable Gastric Band (VAD-BAND) for treatment of morbid obesity, and potential consideration for future cardiac transplantation. An open adjustable gastric band was placed through an abdominal incision, followed by placement of the ventricular assist device.

## Results

Both patients underwent open placement of the adjustable gastric band prior to placement of the VAD (Table 1). Patient 1 was a 24 year old male with a body mass index (BMI) of 46.6 kg/m^2^ admitted in cardiogenic shock with severe non-ischemic cardiomyopathy (New York Functional Class IV, Left Ventricular Ejection Fraction 15.3%) to the intensive care unit. The patient concurrently underwent the VAD-BAND procedure with no peri-operative complications. At 11 months outpatient follow-up the patient had clinically improved with a BMI of 34.2 kg/m^2^. Patient 2 was a 36 year old male with a body mass index of 43.7 kg/m^2^ admitted in cardiogenic shock with severe non-ischemic cardiomyopathy (New York Functional Class IV, Left Ventricular Ejection Fraction 17.1%) to the intensive care unit. Patient 2 underwent placement of a VAD-BAND and at 4 months post-operation, the patient was stalwart clinically with a BMI of 34.8 kg/m^2^. Additional operative for BAND placement was 36 and 29 minutes and did not affect short-term post-operative outcomes. Intermediate follow-up demonstrates a successful weight reduction and improvement in cardiac parameters (Left Ventricle Index Dimension-LVID). Both patients are now under consideration for cardiac transplantation.

**Table 1:** Pre- and Post-operative Body Mass Index in Patients

	Age (years)	Pre-op LVID-Diastolic (normal: 3.8 - 5.7 cm)	Pre-op LVID- Systolic (normal: 2.2 - 4 cm)	Pre-op BMI	Follow-up time (months)	Post-op LVID-Diastolic	Post-op LVID-Systolic	Post-op BMI
1	24	7.4 cm	6.0 cm	46.6	14	4.8 cm	4.0 cm	34.71
2	36	6.8 cm	5.6 cm	43.7	5	4.5 cm	3.6 cm	38.55

BMI: body mass index; LVID: Left Ventricle Index Dimension; Pre-op: preoperative; Post-op: postoperative.

## Discussion

This study reports the first two cases of successful VAD-BAND placement in morbidly obese patients with end-stage heart failure. Considering that the VAD generator is placed into the abdominal cavity, placement of the adjustable gastric band may be preformed efficiently and safely in this patient population.

The demographic of the North American patient continues to change, with an overall trend toward increased BMI [2]. Morbidly obese patients with severe, refractory end-stage heart disease have limited treatment options. These include maximal medical therapy, insertion of VADs as a bridge to cardiac transplantation or cardiac transplantation itself [8]. According to Dhesi et al, insertion of VAD alone produced significant moderate weight loss, resulting improved BMI at 6 months post-insertion (36.1 kg/m^2^ to 31.8 kg/m^2^) [9]. However, these authors observed no significant change with maximal medical management alone. Though these findings are positive, there is a paucity of evidence in morbidly obese patients with end-stage heart disease. Cardiac transplantation is typically contraindicated in these patients secondary to limited organ availability and increased mortality and morbidity rates [7]. Contrastingly, insertion of VAD has been shown to be successful in obese patients without increased mortality comparatively [10]. Therefore, to decrease the mortality risk in morbidly obese patients, the addition of an adjustable gastric band was considered. As a primarily restrictive procedure, that is reversible, and does not alter gastrointestinal continuity, it has been shown to produce marked weight loss in morbidly obese patients without heart failure [11]. The successful placement of the VAD-BAND in these patients suggest that the VAD-BAND may be reasonable option in morbidly obese patients, that otherwise may not be candidates for transplantation.

There are several limitations must be considered. Firstly, we have only two patients in our series. Larger patient numbers in the form of a clinical trial may be a safe approach to a new therapeutic concept. Secondly, the follow-up of our two patients is relatively short. However, the marked weight loss seen in both patients, suggests that the VAD-BAND may represents a promising advancement in both managing this patient population and fostering the potential for future cardiac transplantation for an otherwise terminal condition.

### Conclusions

In conclusion, concurrent placement of a VAD-BAND may be a safe and viable option for morbidly obese patients with end-stage heart disease. Further research is needed to define indications and future clinical practice.
